# Fatty Aldehydes in Cyanobacteria Are a Metabolically Flexible Precursor for a Diversity of Biofuel Products

**DOI:** 10.1371/journal.pone.0058307

**Published:** 2013-03-11

**Authors:** Brett K. Kaiser, Michael Carleton, Jason W. Hickman, Cameron Miller, David Lawson, Mark Budde, Paul Warrener, Angel Paredes, Srinivas Mullapudi, Patricia Navarro, Fred Cross, James M. Roberts

**Affiliations:** 1 Matrix Genetics, Seattle, Washington, United States of America; 2 Department of Biochemistry, University of Washington, Seattle, Washington, United States of America; 3 Department of Pathology and Laboratory Medicine, University of Texas Health Science Center, Houston, Texas, United States of America; 4 The Rockefeller University, New York, New York, United States of America; 5 Division of Basic Sciences, Fred Hutchinson Cancer Research Center, Seattle, Washington, United States of America; Max Delbrueck Center for Molecular Medicine, Germany

## Abstract

We describe how pathway engineering can be used to convert a single intermediate derived from lipid biosynthesis, fatty aldehydes, into a variety of biofuel precursors including alkanes, free fatty acids and wax esters. In cyanobacteria, long-chain acyl-ACPs can be reduced to fatty aldehydes, and then decarbonylated to alkanes. We discovered a cyanobacteria class-3 aldehyde-dehydrogenase, AldE, that was necessary and sufficient to instead oxidize fatty aldehyde precursors into fatty acids. Overexpression of enzymes in this pathway resulted in production of 50 to 100 fold more fatty acids than alkanes, and the fatty acids were secreted from the cell. Co-expression of acyl-ACP reductase, an alcohol-dehydrogenase and a wax-ester-synthase resulted in a third fate for fatty aldehydes: conversion to wax esters, which accumulated as intracellular lipid bodies. Conversion of acyl-ACP to fatty acids using endogenous cyanobacterial enzymes may allow biofuel production without transgenesis.

## Introduction

As photoautotrophs with minimal nutrient requirements, cyanobacteria offer an attractive platform for carbon-neutral production of biofuel products [1]. The fatty acid biosynthesis (FAS) pathway, which produces long-chain carbon intermediates that are polymerized on acyl carrier protein (ACP) [2], holds particular promise as a target for metabolic engineering of biofuel precursors [3]. FAS is tightly regulated by feedback inhibition of at least three enzymatic activities–acetyl CoA carboxylase (ACCase), 3-oxoacyl-ACP-synthase (FabH), enoyl-ACP reductase (FabI)–by long chain acyl-ACP [2], [4], [5], [6]. Therefore, efficient biofuel production requires diversion of FAS-derived intermediates into commercially important products without triggering such feedback inhibition. One successful strategy has been overexpression of thioesterase enzymes, which cleave acyl-ACP to yield ACP and free fatty acid [7], [8], [9]. Overexpression of plant thioesterases in *Synechocystis* sp. PCC 6803 results in a significant net accumulation of free fatty acid [10]. Moreover, free fatty acids are excreted, suggesting the possibility of a continual production-harvest system [10].

As an alternative approach, in some organisms wax esters are formed by the condensation of a fatty acid and a fatty alcohol, and are stored internally as lipid bodies [11]. Exogenous expression of the enzymes that catalyze this reaction–wax ester synthase/diacylglycerol acyl transferases (WS/DGAT) [12]– in *E. coli* promotes wax ester production [13], but has yet to be demonstrated in cyanobacteria. In *E. coli* wax ester production was facilitated by overexpression of fatty acyl-coA reductase (FAR; [13]), which converts acyl-coA into a fatty alcohol [14], [15], [16]. Most or all cyanobacterial species appear not to produce acyl-coA–evidenced by the lack of an obvious acyl-coA ligase homolog and inability to incorporate exogenous fatty acids [17]–thus engineering cyanobacteria to produce wax esters will require a modified approach to what has been demonstrated in other fermentative bacteria.

**Figure 1:**
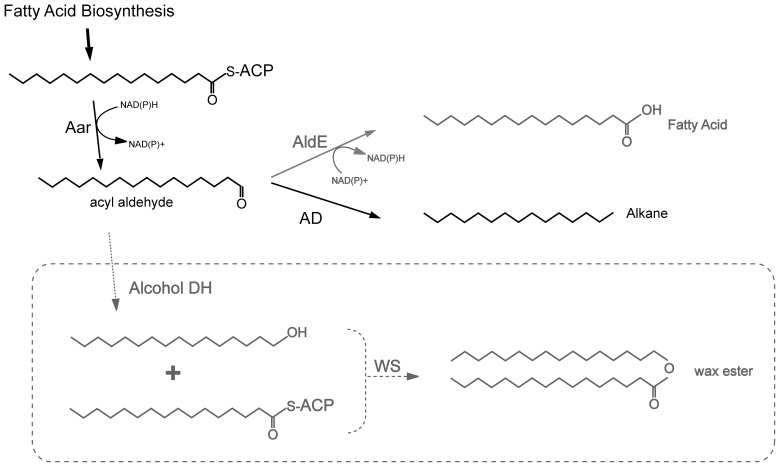
Alternative fates of the acyl aldehyde intermediate produced by Aar. Acyl-ACP reductase (Aar) converts the end-product of fatty acid biosynthesis, acyl-ACP, into an acyl aldehyde. This intermediate can be converted into alkanes or free fatty acids by the endogenously encoded aldehdye decarbonylase (AD) and AldE enzymes, respectively; or into wax esters by co-expression of alcohol dehydrogenase and wax ester synthase (WS) transgenes. Previously described pathways in *S. elongatus* are in black; pathways described in this paper are in gray.

Alkanes are also attractive biofuels because of their minimal downstream processing requirements. Certain species of cyanobacteria naturally produce alkanes via a unique two-enzyme pathway that is encoded in a highly conserved operon [18], [19]. The first enzyme in this pathway, acyl-ACP reductase (Aar), catalyzes breakdown of acyl-ACP to a fatty aldehyde and ACP [18]. An aldehyde decarbonylase (AD) then converts the aldehyde intermediate into an alkane. Co-expression in *E. coli* of the cyanobacterial enzymes derived from *Synecchococcus elongatus* PCC 7942 yields alkanes. However, most Aar-generated aldehydes are not converted to alkanes, but to fatty alcohols by endogenous *E. coli* alcohol dehydrogenases [18]. Competition with these enzymes, and intrinsic inefficiency of AD [20], [21], [22], [23], may limit large-scale production of alkanes. Nevertheless, the fatty aldehyde in this pathway could serve as intermediate for other biofuel precursors (Fig. 1), and most likely will not trigger feedback inhibition of FAS. We have therefore explored photoautotrophic production in cyanobacteria of diverse biofuel precursors from fatty aldehydes: alkanes, fatty acids and wax esters.

## Results

### Increased Fatty Acid Production upon Aar Overexpression

**Figure 2:**
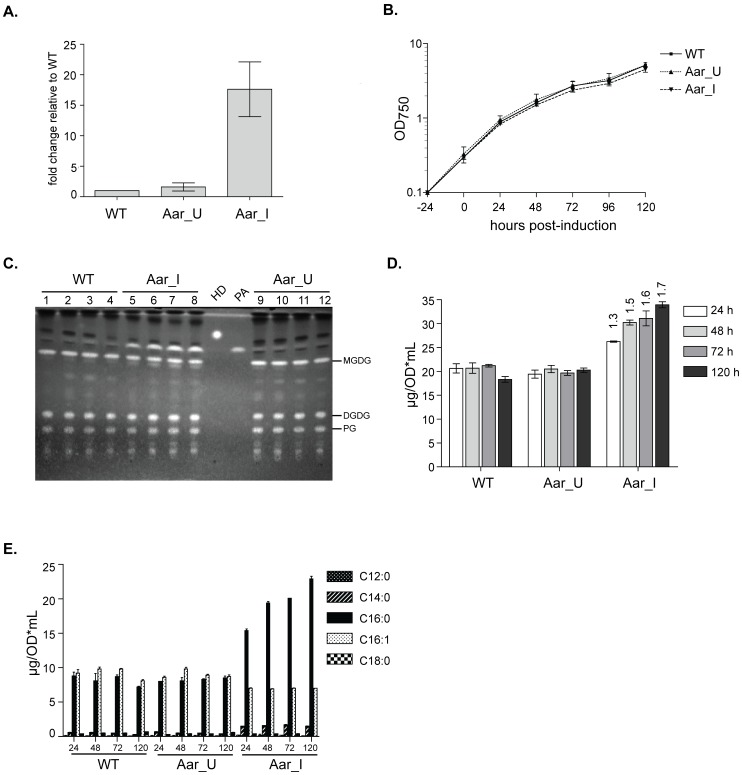
Overexpression of Aar (encoded by orf1594) leads to increased free fatty acid production in *S. elongatus* PCC 7942. **A.** qPCR analysis of *aar* expression levels. WT and IPTG-inducible Aar strains were grown as described in materials and methods, and induced with 1 mM IPTG at t = 0. “I” indicates induction; “U” indicates uninduced. Total cellular RNA was obtained from all samples at 24 hour post-induction. RNA was converted to cDNA and relative *aar* transcript levels were assessed by quantitative PCR. RNA input for each sample was normalized to *rnpB*, and *aar* expression is presented as the fold change in expression compared to endogenous *aar* from a WT strain. The data is the average of 6 independent experiments; error bars are the standard deviation. **B.** Growth curves of the indicated strains (log OD_750_ vs. time). The WT strain is the average of 6 independent experiments; Aar_U and Aar_I are the average of 10 independent experiments. Error bars are the standard deviations. **C.** 0.5 OD_750_-equivalents from samples collected at 24, 48, 72 and 120 hours post-induction were separated by thin layer chromatography (TLC) using polar solvent for the mobile phase. 10 µg of hexadecanol (HD) and 5 µg of palmitic acid (PA; C16∶0 free fatty acid) were included as standards. MGDG: monogalactosyldiacylglycerol; DGDG: digalactosyldiacylglycerol; PG: phosphatidylglycerol. **D.** Total fatty acid methyl esters (FAMES) assessed by GC. Each timepoint is the average of three measurements; error bars are standard deviations. The numbers above the Aar_I bars indicate the fold-increase of total FAMES compared to WT samples. **E.** Individual constituent FAMES were quantified. The observed increase in total FAMES is due almost entirely to accumulation of C16∶0 free fatty acids (solid black).

Aar-mediated fatty aldehyde production and downstream conversion have not yet been characterized in cyanobacteria. Therefore, we generated a strain of *S. elongatus* containing an additional copy of Aar (encoded by orf1594) expressed from an IPTG-inducible promoter (Methods; Table S1 in File S1). IPTG induction resulted in 10–20 fold higher expression of *aar* transcript 24 hours post-induction relative to endogenous *aar* levels in a WT strain (Fig. 2A). Induction of Aar had no significant effect on population doubling times, compared to WT or uninduced strains (Fig. 2B). However, Aar-induced cultures accumulated a white precipitate, similar to fatty acids secreted by thioesterase-expressing strains ([10]; our observations). Analysis by thin layer chromatography showed that Aar induction caused a large increase in a band that co-migrated with palmitic acid (C16∶0; Fig. 2C) but not with hexadecanol, the predominant species that accumulated in *E. coli* upon Aar induction [18]. By gas chromatography (GC), Aar-induced samples contained a large increase in a peak that co-eluted with a C16∶0 standard. These results demonstrated that Aar overexpression resulted in fatty acid accumulation.

Quantitation of transesterifiable fatty acid methyl esters (FAMES) from whole culture (i.e. cells and medium) demonstrated that Aar induction increased FAMES production by an initial rate of ∼5 µg/OD*ml/day (Fig. 2D). Production continued for at least 5 days, at a declining rate. At 5 days post-induction, FAMES were 1.7-fold higher than WT or uninduced samples (Fig. 2D). This increase was almost entirely from palmitic acid (Fig. 2E). The increase in FAMES upon Aar overexpression implied fatty acid production in excess over the amounts required for membrane lipid biosynthesis. This redirection of metabolism occurs with remarkably little toxicity, most likely because of the efficient extracellular accumulation of free fatty acid from the cells. These results have implications for the potential use of this system for biofuel production.

We also quantified pentadecane and heptadecane in *S. elongatus* produced by Aar induction (Figure S1 in File S1). Aar overexpression increased pentadecane ∼2-fold, but the overall amount was substantially less than the free fatty acids produced. At 3 days post-induction total fatty acids were 13 µg/OD*mL higher relative to an uninduced strain, whereas the levels of pentadecane were only increased 0.1 ug/OD*mL. Heptadecane was not significantly increased, consistent with Aar primarily utilizing C16 acyl-ACP as a substrate. Thus *S. elongatus* preferentially converted the acyl aldehyde intermediate produced by Aar to free fatty acid rather than alkanes.

### AldE, an Aldehyde Dehydrogenase Responsible for Fatty Acid Production upon Aar Overexpression

The robust production of free fatty acids by Aar overexpresion suggested the existence of an aldehyde dehydrogenase that would oxidize the free fatty aldehyde intermediate (Fig. 1). We identified a class 3 aldehyde dehydrogenase ortholog encoded by synpcc7942_0489 (orf0489) in the *S. elongatus* PCC7942 genome. Orthologues with high sequence identity (>50%) are present in mammals, plants and bacteria, and some can reduce long chain aliphatic aldehydes [24], [25], [26]. Clear homologues of orf0489 are present in many species of cyanobacteria, with the highest sequence identities reaching ∼55%.

**Figure 3:**
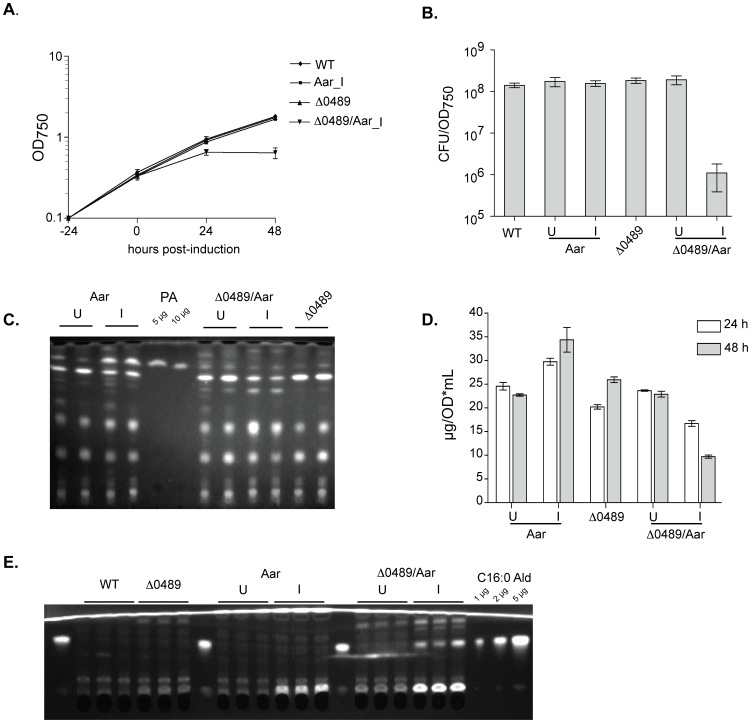
orf0489 is required for Aar-induced FFA production and to mitigate Aar toxicity. **A.** Growth curve of the indicated strains (log OD_750_ vs time). For clarity, only cultures induced with IPTG are shown. There was no significant difference in growth rate in any of the uninduced strains compared to WT. Values are the average +/− SD of three independent experiments. **B.** Colony forming units per OD_750_ (CFU/OD_750_) of the indicated strains were determined 48 h post-induction. WT is the average of five independent experiments; the remaining strains are the average of three independent experiments. Error bars are the standard deviation. **C.** 0.5 OD_750_-equivalents from samples collected 24 and 48 hours post-induction were resolved by TLC using polar solvents (described in Materials and Methods). PA: palmitic acid. **D.** Total FAMES 24 and 48 h post-induction of the indicated strains. **E.** In a separate experiment from A-D, samples from biological triplicates were collected 24 h post-induction, and 1 OD_750_-equivalents were resolved on TLC using non-polar solvents (see Materials and Methods). A C16∶0 fatty aldehyde standard (i.e. hexadecanal) was included between lanes and on the flanking lanes.

To determine whether orf0489 converted Aar-induced fatty aldehydes to free fatty acids, we deleted orf0489 from WT and Aar-inducible strains. Deletion of orf0489 in a WT background had no significant effect on growth (Fig. 3A). However, induction of Aar in the Δ0489 background resulted in cessation of growth and a 2–3 log decrease in viability (measured as CFU/OD_750_) at 48 hours post-induction (Fig. 3A, B). Further, the Aar/Δ0489 strain failed to accumulate palmitic acid (Fig. 3C, D), but did accumulate hexadecanal (Fig. 3E), conclusively demostrating that orf0489 was required for fatty aldehdye conversion to free fatty acids. The lethality observed in the induced Aar/Δ0489 strain was most likely caused by fatty aldehyde accumulation. We analyzed samples on thin layer chromatography (TLC) using two different solvents that differed in the degree of hydrophobicity (“polar” and “non-polar”, Fig. 3C and 3E). With both solvents, several unidentified bands appeared in the induced Aar/Δ0489 strain in addition to hexadecanal (Fig. 3C and 3E). One of these bands also appeared in the Δ0489 strain (Fig. 3E). It was possible that this molecule was the physiological substrate of orf0489, although its accumulation did not seem to adversely affect growth of the Δ0489 strain. The identity of these bands is the subject of ongoing investigations. These results established that orf0489 was required for free fatty acid production induced by Aar overexpression, as well as to mitigate toxicity caused by Aar. In accordance with standard nomenclature, we have named orf0489 *aldE*.

We next expressed and affinity purified 6Xhis-tagged AldE from *E. coli* in order to analyze its *in vitro* biochemical properties (Figure S2 in File S1). The elution profile of purified h_6_-AldE on size-exclusion chromatography was consistent with a dimer, as is the case for other class 3 aldehyde dehydrogenases [27], [28]. We tested the ability of h_6_-AldE to convert aldehyde substrates (containing 4 to 16 carbons) to fatty acids (Methods; Table 1). h_6_-AldE demonstrated the highest efficiency (i.e. k_cat_/K_m_) towards medium chain aldehdyes (octanal, decanal and dodecyl aldehyde) but was also able to utilize shorter and longer chain lengths; K_m_s ranged from 10–50 µM. These enzymatic features were very similar to the human class 3 aldehyde dehydrogenase [24], [26]. The turnover of h_6_-AldE ranged from ∼450–800 min^−1^ for various substrates. In contrast, the maximum reported turnover for aldehyde decarbonylase was ∼18 min^−1^ for heptanal [23]. These *in vitro* results correlate with the preferential accumulation of fatty acids over alkanes by induction of Aar *in vivo*. In sum, we showed that AldE was both necessary (*in vivo*) and sufficient (*in vitro*) for the synthesis of fatty acids from fatty aldehydes.

**Table 1:** Kinetic parameters of h_6_-AldE from *S. elongatus* PCC7942^1^.

Substrate	K_m_ (µM)	k_cat_ (min^−1^)	k_cat_/K_m_ (min^−1^µM^−1^)
Formaldehyde	undetectable		
Butyraldehyde	189.4±25.1	459.2±35.5	2.4±0.2
Hexanal	19.3±4.4	510.5±24.2	26.5±0.2
Octanal	15.4±5.2	810.1±63.2	52.5±0.3
Decanal	7.6±3.1	724.3±65.0	95.6±0.4
Dodecyl aldehyde	11.4±2.4	557.5±74.9	49.1±0.2
Hexadecenal	38.1±10.5	647.8±37.4	17.0±0.3

1 Results are from three independent experiments and are expressed as average ± standard deviation.

### Aar Levels were Limiting for Fatty Acid Production

**Figure 4:**
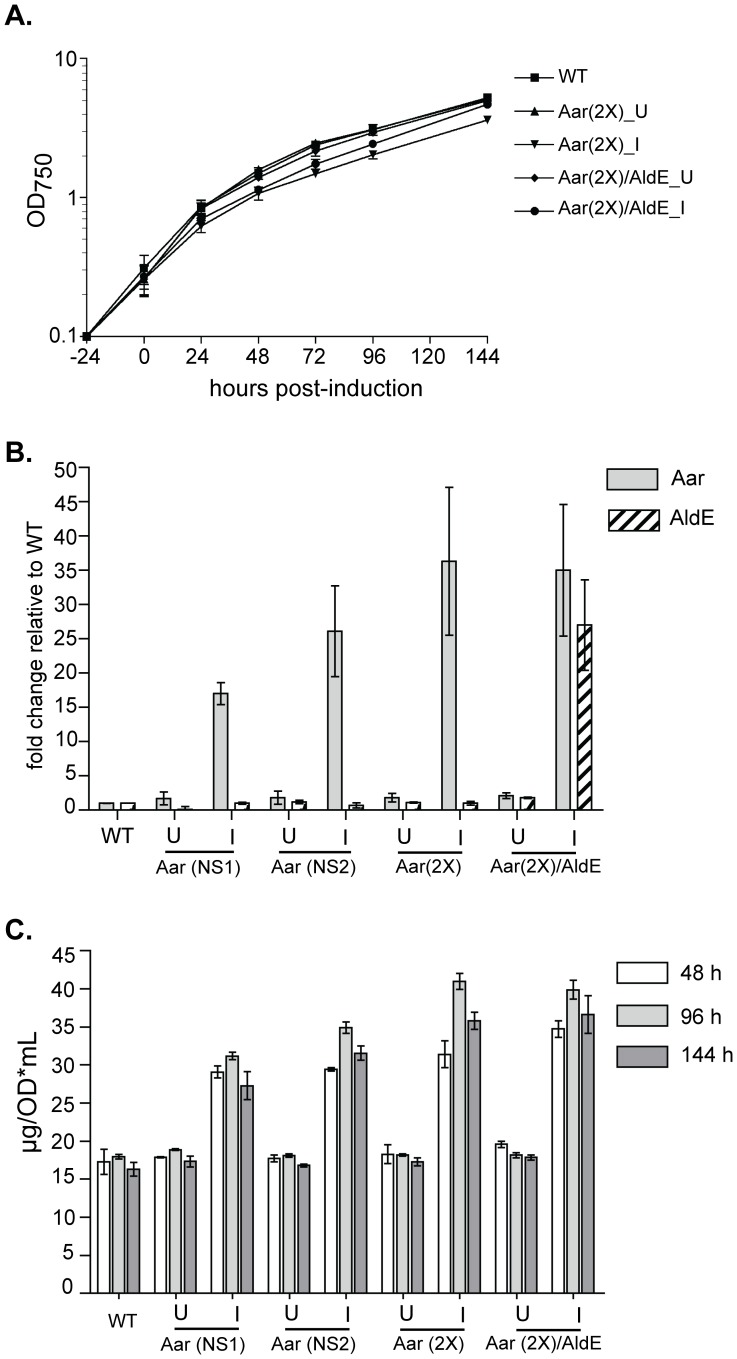
Overexpression of two copies of Aar leads to increased FFA production, but at a growth cost that is alleviated by AldE overexpression. Strains were constructed containing a single copy of Aar in neutral site 1 (NS1) or neutral site 2 (NS2); two copies of Aar (2X); or two copies of Aar and an additional copy of AldE (“Aar(2X)/AldE”). **A.** Growth curve of the indicated strains. For clarity only the Aar (2X) and Aar(2X)/AldE strains are presented; Aar(NS1) and Aar(NS2) strains were processed in parallel for FAMES analysis. **B.** Transcript levels of *aar* and *aldE* were assessed by qPCR from samples collected 24 hours post-induction and are presented as the fold increase relative to endogenous expression of each gene from a WT strain. The data are the average of three experiments +/− SD. **C.** Total FAMES of the indicated strains.

In order to test whether Aar levels were limiting for fatty acid production, we created a strain expressing two copies of Aar (“Aar(2X)”), with one copy each expressed from NS1 and NS2. Strains expressing a single copy of Aar from these neutral sites (i.e. Aar (NS1) and Aar (NS2)) had similar growth rates, Aar transcript production (Fig. 4B) and FAMES production (Fig. 4C). The Aar (2X) strain produced ∼1.5-fold more Aar transcript than corresponding strains containing either single copy of Aar (Fig. 4B), with little change in abundance of endogenous AldE transcript (Fig. 4B). Induction of Aar(2X) slowed growth (Fig. 4A), and further increased fatty acid production: 5–10 µg/OD*mL/day greater in the Aar(2X) than the Aar (1X) strains (Fig. 4C).

AldE overexpression (∼30 fold higher than endogenous levels; Fig. 4B) mostly rescued the Aar(2X) growth defect (Fig. 4A), suggesting that induction of Aar(2X) produced fatty aldehydes in amounts greater than could be converted to fatty acids by endogenous AldE enzyme levels. However, despite improving viability of Aar-overexpressors, AldE overexpression did not significantly increase the rate of fatty acid production. This suggested that relatively small amounts of fatty aldehydes were responsible for the decreased viability caused by induction of Aar(2X). The Aar(2X)/AldE double over-expressor thus exhibits significantly increased total lipid production, without a notable reduction in viability or growth rate.

### Production of Wax Esters

**Figure 5:**
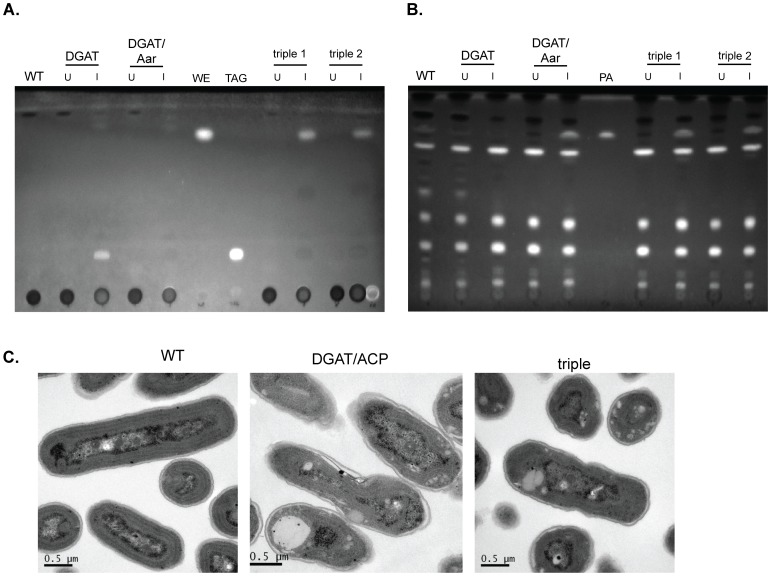
Aar-dependent production of wax esters in PCC7942. Strains containing the WS/DGAT enzyme from *A. baylyii* (“DGAT”); DGAT and Aar; DGAT, Aar and slr1192 (“triple 1); or DGAT, Aar and ACIAD3612 (“triple 2″) were analyzed 24 hours post-induction by TLC using nonpolar solvents to resolve WE and TAG (**A**) or polar solvents to resolve fatty acids (**B**). slr1192 and ACIAD3612 are long-chain aldehyde dehydrogenases from *Synechocystis* and *A. baylyii*, respectively. 0.5- OD_750_ equivalents were loaded per well. WE: wax ester; TAG: triacylglycerol; PA: palmitic acid. **C.** Electron micrographs of the indicated strains 24 hours post-induction. Lipid bodies are present in DGAT-containing samples and appear as white globules.

Neutral lipids, including wax esters and triacylglycerols (TAG), are potential biofuel precursors and naturally-occurring energy storage compounds in some species of bacteria, plants and eukaryotic algae. Cyanobacteria, however, do not naturally accumulate either TAG or wax esters, and there are no reports of pathway engineering being used to make these products. Wax esters and TAG synthesis can be catalyzed by a prokaryotic enzyme (WS/DGAT) that condenses long acyl chains with fatty alcohols or diacyl glycerol (DAG) to form wax esters or TAG’s [12]. The majority of activated fatty acid intermediates that could be used for TAG and wax ester synthesis are thioesters linked to either coenzyme A or ACP. acyl-CoA is predominantly utilized by enzymes dedicated to energy production (e.g. by the ß-oxidation pathway), or energy storage (e.g. neutral lipid synthesis). To date, the metabolic engineering of neutral lipids in other bacterial species has taken advantage of acyl-CoA specific enzymes (e.g., [13]). In cyanobacteria, most if not all fatty acid intermediates are in the form of acyl-ACP, and are dedicated primarily to lipid biosynthesis, lipid A formation and alkane production. It was therefore unclear as to whether WS/DGAT could function in cyanobacteria. We first tested whether WS/DGAT was active in cyanobacteria by overexpressing WS/DGAT from *Acinetobacter* sp. ADP1 (“aDGAT” [12]) in *S. elongatus.* Overexpression of aDGAT resulted in TAG formation (Fig. 5A, C).

Based on this observation, we asked whether we could take advantage of Aar activity to engineer a pathway for the formation of wax esters into *S. elongatus*: the aldehyde intermediate produced by Aar could be further reduced to a fatty alcohol, which could then be condensed with a second acyl-ACP by aDGAT to generate a wax ester (Fig. 1). To test this, we co-expressed Aar with both a long-chain alcohol dehydrogenase from *Synechocystis* sp PCC 6803 (slr1192 [29]) and aDGAT. This resulted in wax ester production (Fig. 5A). A related alcohol dehydrogenase *(*ACIAD3612 from *A. bayli*; 48% identity to slr1192) also produced wax esters when coexpressed with Aar and aDGAT (Fig. 5; “triple 2”). These strains also produced free fatty acids (Fig. 5B), as would be expected due to endogenous AldE activity. In the wax ester-producing strains no TAG was detectable, even though overexpression of aDGAT alone resulted in TAG formation (Fig. 5A). These results indicated that: 1) the combined expression of Aar and alcohol dehydrogenase must have resulted in the production of the fatty alcohol substrate for aDGAT, although this was not explicitly measured; and 2) the wax ester synthase activity of aDGAT predominated over its diacylglycerol acyltransferase activity, consistent with previously characterized substrate specificity of aDGAT [12].

Bacteria that naturally produce TAG- and wax esters accumulate lipid bodies [11]. In our engineered TAG and WE- producing *S. elongatus*, we observed lipid bodies (Fig. 5C), frequently associated with disrupted thylakoid membranes. Accumulation of TAGs or wax esters resulted in loss of chlorophyll a within 24 hours, and a 4-log viability drop by 48 hours. This contrasted with the full viability of the FFA-secreting Aar-overexpressors, probably because FFA secretion prevented intracellular damage such as thylakoid membrane disruption.

## Discussion

Cyanobacteria have the potential to reduce demand for fossil fuels because of their neutral carbon footprint, their ability to produce numerous biofuel related products, and their facile genetics [1]. Here we describe how fatty aldehydes shunted from lipid biosynthesis by Aar can act as a multifunctional precursor for the production of fatty acids and wax esters, in addition to alkanes [18]. Alkane production in cyanobacteria occurs by the sequential reduction and decarbonylation of acyl-ACP. Expression of the cyanobacterial enzymes that carry out these reactions–Aar and AD– in *E. coli* resulted in some alkane production (penta- and heptadecane), but at far lower levels than other products, such as hexadecanol [18]. In *S. elongatus*, Aar overexpression similarly resulted in only a low level of alkane production (Figure S1 in File S1), but rather than fatty alcohols, the major product was free fatty acids, present at 50–100-fold the yield of alkanes. We identified the endogenous aldehyde dehydrogenase - AldE (orf0489) - that oxidizes the Aar-produced aldehyde intermediate. AldE is a class 3 aldehyde dehydrogenase, a widely represented family in eukaryotes and prokaryotes [30]. Fatty acids offer a significant advantage as a biofuel precursor because they are excreted from the cell ([10], our own observations) and can be continuously harvested, but up to now their production has only been achieved by transgenic overexpression of foreign acyl-ACP thioesterases [10]. Production of free fatty acids by sequential action of Aar and AldE demonstrated here is of particular interest because it operates via endogenous cyanobacterial genes, and therefore may provide a non-transgenic solution to algal biofuel production. Free fatty acid production by Aar overexpression results in at least 80% the levels we have routinely observed upon overexpression of plant thioesterases in *S. elongatus* (data not shown).

Although endogenous AldE efficiently oxidizes hexadecanal produced by overexpression of Aar to palmitic acid, this may not be its physiological substrate. Consistent with this possibility, we detected a relatively prominent band in the ΔAldE strain using nonpolar solvents on TLC (Fig. 3E). In addition, h_6_-AldE had higher specific activity *in vitro* against medium chain aldehydes compared to long-chain aldehydes.

Homologs of AldE are widely represented in bacteria with relatively high sequence similarity (40–50% at the amino acid level). Only two of these–alkH from *P. oleovorans* (44% % identity; [25]) and coniferyl aldehyde dehydrogenase from *Pseudomonas* sp. strain HR199 (43% [28])–have been characterized, and likely have functions distinct from AldE. A clue to AldE function could come from human and rat class 3 aldehyde dehydrogenases, which share high sequence homology with AldE (∼50% identity) and have similar enzymatic profiles [24], [26], [31], [32]. These enzymes have been postulated to remove toxic aldehyde intermediates that are breakdown products of lipid peroxides, which are generated from oxidative damage to polyunsaturated fatty acids (PUFA) [33]. The importance eliminating toxic fatty aldehyde intermediates is underscored by Sjogren-Larsson syndrome, a neurological syndrome that is caused by a mutation in the human class 3 ALDH [34]. In *A. thaliana*, expression of the ALD3H1 (40% identity to AldE) conferred resistance to numerous stresses including peroxide treatment, perhaps also pointing to a role in removing toxic lipid peroxide products [33], [35]. It should be noted that under normal laboratory growth conditions, *S. elongatus* PCC 7942 contains very low levels of PUFA (the majority of its lipids are C16∶0 and C16∶1, in nearly equivalent amounts); thus it is not obvious that *S. elongatus* would generate significant amounts of lipid peroxides, and hence aldehyde breakdown products. *S. elongatus* and other species of cyanobacteria were recently shown to encode acyl-ACP synthase (Aas), which converts free fatty acids to acyl-ACP and has been proposed to be required for free fatty acid recycling [17]. AldE may act in concert with Aas in recycling membrane lipids.

We also explored the possibility of producing neutral lipids (TAG and wax esters) in *S. elongatus*. Expression of the dual function enzyme WS/DGAT alone resulted in TAG production. To our knowledge this is the first demonstration that WS/DGAT enzymes are active in cyanobacteria, and the first demonstration of TAG production in cyanobacteria by any method. We then induced wax ester formation by co-expressing three enzymes - Aar, a long-chain alcohol dehydrogenase and WS/DGAT (Figures 1 and 5). Production of either TAG or wax esters resulted in lipid body formation, as has been seen in other organisms that produce neutral lipids [11]. However, the generation of either product resulted in toxicity, which could be caused by the lipid bodies themselves, promiscuous substrate usage by the WS/DGAT, or substrate depletion. Production of neutral lipids in cyanobacteria as a source of biofuels will require engineering around this toxicity.

Although photosynthetic microbes have appeal as potential biofuel crops due to rapid generation times and high efficiencies of photosynthesis, a natural microbial isolate might not initially be suitable. Free-living microbes have evolved to maximize increase of their genetic material through time. Any production of fats in excess of that needed to maximize propagation of their genome is evolutionary ‘wasted effort’. Nevertheless, in certain circumstances algae or cyanobacteria store large amounts of carbohydrate or fat as energy reserves, which led to the proposal that they could be useful as biofuel crops [36]. However, accumulation of these carbon stores is currently limited to nutrient-restricted regimes, for use at a later time when nutrient availability is higher. This regulatory aspect of fat storage has the paradoxical result that maximum lipid accumulation actually occurs during rapid growth in nutrient-replete conditions, even though storage lipid is relatively low under these conditions - the accumulated fat is simply a part of the minimal biomass (e.g. thylakoid and cytoplasmic membranes) required for cell reproduction [36]. An ideal biofuel crop, in contrast, would produce amounts of fat in great excess over what is needed for cell reproduction, even under nutrient-replete conditions. Thus, conversion of a natural isolate to a biofuel crop requires a substantial redirection of carbon flow from biomass to fuel precursors; in the case of Aar-overexpressors, secreted free fatty acids. This requires engineering in opposition to billions of years of natural selection.

If a free-living microbe is evolutionarily optimized for production of new cells, then its most rapid potential rate of (carbon-based) biofuel synthesis can be approximated by the rate of carbon fixation into cellular biomass under ideal conditions. This is likely to be near the maximum since biomass growth is close, though not identical, to the property under direct natural selection. In our standard conditions (see Methods) the maximum rate of *S. elongatus* biomass accumulation is in the range of 0.3–0.6 grams dry weight/liter/day. Approximately 50% of this biomass is carbon, and therefore the estimated maximum conversion rate of fixed carbon into biofuels is 0.15–0.3 grams/liter/day. This could scale to a theoretical maximum of ten to twenty thousand gallons oil/acre/year in a production setting if 100% of fixed carbon were captured as a fatty biofuel precursor. This calculation, which emerges from straightforward biological considerations and empirical observations of cyanobacteria growth rates, closely approximates optima derived by thermodynamic analysis of photosynthetic efficiencies and other theoretical considerations [37].

An informative benchmark is our Aar-expressing *S. elongatus* strain. It secretes free fatty acids at a rate of 15 µg/OD*mL/day, which represents the diversion of 10% of photosynthetically harvested carbon into the biosynthesis of new lipids. This would predict a rate of oil synthesis (excluding membrane lipids) of 1,000 gallons/acre/year, which is about twice the yield of palm oil [38], the most productive terrestrial oil seed crop.

These calculations illustrate that the yields of cyanobacterial oil could be substantially improved by slowing (or conditionally blocking) cell duplication and attendant accumulation of biomass in favor of redirecting fixed carbon into biofuel precursors [10]. This is a central feature of domesticated terrestrial crops, in which the allometric proportion of seed or fruit to other plant organs has drastically increased compared to wild relatives. In this regard, it is promising that strains overexpressing Aar have already demonstrated a surprising degree of metabolic flexibility in the deployment of fixed carbon; fatty acids are produced and secreted at a rate equal to that of membrane lipid biosynthesis. We have shown that the limiting step in fatty acid production is the production of fatty aldehydes by Aar. Increasing carbon flow through upstream reactions might further enhance the output of biofuel precursors.

Implementation of this strategy will be hindered by endogenous regulatory systems that evolved to prevent the uncoupling of growth and photosynthesis. For example, a cell sending all of its fixed carbon into biofuel might experience a starvation or fixed carbon deficit signal, resulting in activation of shutdown responses, which dismantle their photosystems almost entirely. Thus, laboratory construction of a biofuel-producing microbe will require detailed understanding of the global physiology of the organism through application of systems biology approaches. The capacity to remodel metabolism requires a highly malleable genetic system. It is for this reason that cyanobacteria, for which a full range of powerful molecular genetic technology already exists, are currently the only plausible sources of an optimized biofuel crop.

## Materials and Methods

### Reagents

Unsaturated lipid standards for GC were obtained from Nu-Chek Prep. All other chemicals were from Sigma Aldrich.

### Protein Purification and Enzymatic Assays

*aldE* (orf0489) was subcloned into a pET22b+ expression vector modified to contain an N-terminal 6XHis tag, expressed in BL21(DE3)RIL *E. coli*, and purified by metal affinity chromatography as described [39]. The eluted fraction was then loaded on a HiLoad 16/60 SuperDex200 gel filtration column (Sigma) equilibrated in 150 mM NaCl/50 mM Tris, 7.5/1 mM EDTA. The peak fractions were concentrated in a 30,000 MWCO Centricon (Millipore) to ∼1 mg/mL and immediately used in enzyme assays, or supplemented with glycerol to 10%, flash frozen and stored at −80°C. Final yields of h_6_-AldE were ∼2 mg per L of culture.

### Enzyme Assay

The activity of h_6_-AldE was assayed at 23°C by monitoring the change in absorbance of NADH at 340 nm using a Spectramax M5 spectrophotomoter in a 1 cm cuvette, collecting readings on kinetic mode every 1 second. The reaction volume was 800 µl and contained 50 mM Tris, 7.5, 150 mM NaCl, 0.05% IGEPAL,1.0 mM NAD^+^, and varying concentrations of aldehyde substrate. Dilutions of aldehydes were made in DMSO. The final concentration of DMSO in the assay was 2% and did not affect enzyme activity. The reactions were initiated by addition of enzyme and initial velocities were determined for the linear range of the reaction. The constants K_m_ and V_max_ were determined by fitting the data using non-linear progression in Prism to the Michaelis-Menten equation Y = (V_max_*X)/(K_m_+X), where X = substrate concentration and Y = velocity; k_cat_ was determined from the equation V_max_ = k_cat_*E_t_, where E_t_ = the concentration of h_6_-AldE. The corresponding values are presented in Table 1 as the mean and standard deviations for three independent measurements. In the absence of enzyme or aldehyde substrate no NADH production (as indicated by a change in 340 nm absorbance) was observed.

### Strains and Growth Conditions

Strains and plasmids used in these studies are presented in Table S1 in File S1. *S. elongatus* strains were grown at 30°C in BG11 media under 50 *u*mol/m^2^/s constant illumination as previously described [40]. Transformations were carried out according to standard protocols [41] by incubating ∼10^8^ cells from a logarithmically growing culture with 100–300 ng of plasmid overnight in the dark. Transformants were plated on BG11 agar plates supplemented with 10 mM bicarbonate and 2 mM sodium thiosulfate and appropriate antibiotics. The following antibiotic concentrations were used in BG11 media and plates: spectinomycyin, 2 µg/mL; streptomycin, 2 µg/mL; kanamycin, 5 µg/mL; gentamycin 4 µg/mL; chloramphenicol 7.5 µg/mL. Spectinomycin and streptomycin were used concurrently for strains containing the Strep^r^ marker. Individual colonies were streaked and patched, and complete segregants were verified by PCR for disruption of the neutral site and presence of the transgene.

Transgenes were PCR amplified from genomic DNA and cloned into recombination vectors. Plasmids for targeting neutral sites 1 and 2 (NS1 and NS2) have previously been described [42]. We generated a new plasmid, pNS4, that is derived from pSP72 (Promega) and contains recombination arms that target the intergenic region between orf’s ORF0893 and 0894; we termed this region neutral site 4 (NS4). The pSP72 plasmid was modified to contain a gentamycin resistance cassette; and a lacI^q^-pTrc promoter that drives transgene expression.

All transgenes were expressed from the pTrc promoter and were induced by 1 mM IPTG. orf1594 (Aar), orf1593 (AD) and orf0489 (AldE) were amplified from *S. elongatus* PCC7942; slr1192 was amplified from *Synechocystis* PCC sp. 6803; and ACIAD3612 was amplified from *Acinetobacter* ADP1. The aDGAT gene from *Acinetobacter* ADP1 was synthesized by DNA2.0 and codon optimized. A null allele of AldE (ΔAldE) was generated by insertion of a chloramphenicol resistance cassette into bp 106–1259 of the AldE open reading frame as described [43].

In a typical induction experiment, strains were inoculated from patches on plates into an 250 mL Erlenmeyer flask containing 40 mL BG11 media plus appropriate antibiotics, grown several days with constant shaking/light at 200 rpm/50 *u*mol/m^2^/s until the OD_750_ reached 1–1.5, diluted back to an OD_750_ of 0.1 without antibiotics, and induced the following day with 1 mM IPTG to start the time course. In all experiments an uninduced culture was processed in parallel for each induced strain. Samples were collected at the indicated times post-induction (1 OD*mL equivalent, triplicate samples for GC, 2 OD*mL for TLC), stored at −80°C and lyophilized before processing for TLC or GC. All growth curves and analysis have been repeated a minimum of three times. For clarity, growth data for uninduced cultures in Figures 3 and 4 are not shown; all of these uninduced strains grew indistinguishably from the WT.

### Cell Viability

CFU assays were performed to determine cell viability by making 10-fold serial dilutions in BG11 media and spotting 10 µl onto BG11 plates (1.5% bacto agar). The plates were incubated at 30°C at ∼100 µE/m^2^/s, and colonies were counted 1 week after plating. The data were normalized to the OD_750_ of each culture and are presented as CFU/OD_750_.

### TLC/GC

For TLC analysis, samples were extracted by the Bligh and Dyer method [44] and resuspended in 2∶1 chloroform:methanol. Typically 0.5 OD_750_-equivalents were loaded on a TLC plate. For separation of neutral lipids (wax esters and TAGs) 90% hexane/10% diethyl ether was used for the mobile phase; for polar lipids the mobile phases was 70 mL chloroform/22 mL methanol/3 mL water. The lipids were visualized by the UV-detectable reagent primuline. For GC analysis, lipid samples were extracted from whole cell cultures via modified Folch extraction [45], Briefly, the dried cell culture samples were spiked with C13∶0 TAG surrogate and resuspended in 1.5 mL of methanol with 4.5% sulfuric acid. The samples were heated for 1 hour at 90°C and cooled to room temperature. 1.5 mL of 1.5% NaCl and 250 µL hexane were then added to each sample. Samples were vortexed and centrifuged at 10,000 rpm for 10 minutes. The transesterified lipids were extracted to the hexane phase, which was removed and injected on the GC. Samples were analyzed on an Agilent 7890 GC using a 10 m×0.25 m×0.25 µm DB-225 capillary column and FID detection. Peak identities were established by retention time comparison to known standards and peak areas were calculated by Agilent Chemstation software. All samples were processed in triplicate and data are presented as mean +/− standard deviation.

### Quantitative PCR

Transcript levels of *aar* (orf1594) and *aldE* (orf0489) were determined 12 to 24 hours post-induction. Total RNA was isolated from 1–10 mL of culture using RNAeasy mini kit (Qiagen) and converted to first-strand cDNA (Invitrogen Superscript II). Primer-probe sets were ordered from Applied Biosystems, and transcript levels were quantified using an ABI PRISM 7900HT sequence detection system. RNA input for each sample was normalized to the housekeeping *rnpB* gene. The expression levels of *aar* and/or *aldE* from the indicated strains were calculated relative to a time-matched, WT strain.

### TEM

4–5 OD_750_ of cells were pelleted and washed in BG11 media, resuspended in fixation buffer (2% glutaraldehyde/0.2M cacodylate, pH 7) for 1 hour at room temperature and stored at 4°C. Samples were processed for TEM according to standard protocols [46], and images were acquired on a JEOL 1200EX electron microscope.

## Supporting Information

File S1 Figure S1: Overexpression of Aar leads to a preferential accumulation of free fatty acids over alkanes. Samples were collected 24, 48 and 72 hours post-induction and analyzed by GC for total FAMES (a), pentadecane (b) and heptadecane (c). Figure S2: Purification of h_6_-AldE (orf0489). AldE containing an N-terminal 6XHis tag was expressed as a soluble protein in *E. coli* and purified by metal affinity and size exclusion chromatrography. The UV trace of the size exclusion chromatography step shows that AldE elutes at a size consistent with a homodimer (AldE is blue trace; green trace is of MW standards), as has been seen for other class 3 aldehyde dehydrogenases [27]. The inset is an SDS-PAGE gel, with the arrow indicating h_6_-AldE. Table S1: Plasmids used in these studies.
(PDF)

## References

[1] Ducat DC, Way JC, Silver PA (2011) Engineering cyanobacteria to generate high-value products. Trends Biotechnol 29: 95–103. 21211860 10.1016/j.tibtech.2010.12.003

[2] Rock CO, Jackowski S (2002) Forty years of bacterial fatty acid synthesis. Biochem Biophys Res Commun 292: 1155–1166. 11969206 10.1006/bbrc.2001.2022

[3] Peralta-Yahya PP, Keasling JD (2010) Advanced biofuel production in microbes. Biotechnol J 5: 147–162. 20084640 10.1002/biot.200900220

[4] Heath RJ, Rock CO (1996) Inhibition of beta-ketoacyl-acyl carrier protein synthase III (FabH) by acyl-acyl carrier protein in Escherichia coli. J Biol Chem 271: 10996–11000. 8631920 10.1074/jbc.271.18.10996

[5] Heath RJ, Rock CO (1996) Regulation of fatty acid elongation and initiation by acyl-acyl carrier protein in Escherichia coli. J Biol Chem 271: 1833–1836. 8567624 10.1074/jbc.271.4.1833

[6] Davis MS, Cronan JE Jr (2001) Inhibition of Escherichia coli acetyl coenzyme A carboxylase by acyl-acyl carrier protein. J Bacteriol 183: 1499–1503. 11157970 10.1128/JB.183.4.1499-1503.2001 PMC95031

[7] Cho H, Cronan JE Jr (1995) Defective export of a periplasmic enzyme disrupts regulation of fatty acid synthesis. J Biol Chem 270: 4216–4219. 7876180 10.1074/jbc.270.9.4216

[8] Jiang P, Cronan JE Jr (1994) Inhibition of fatty acid synthesis in Escherichia coli in the absence of phospholipid synthesis and release of inhibition by thioesterase action. J Bacteriol 176: 2814–2821. 7910602 10.1128/jb.176.10.2814-2821.1994 PMC205434

[9] Voelker TA, Davies HM (1994) Alteration of the specificity and regulation of fatty acid synthesis of Escherichia coli by expression of a plant medium-chain acyl-acyl carrier protein thioesterase. J Bacteriol 176: 7320–7327. 7961504 10.1128/jb.176.23.7320-7327.1994 PMC197121

[10] Liu X, Sheng J, Curtiss R, 3rd (2011) Fatty acid production in genetically modified cyanobacteria. Proc Natl Acad Sci U S A 108: 6899–6904. 21482809 10.1073/pnas.1103014108 PMC3084101

[11] Waltermann M, Steinbuchel A (2005) Neutral lipid bodies in prokaryotes: recent insights into structure, formation, and relationship to eukaryotic lipid depots. J Bacteriol 187: 3607–3619. 15901682 10.1128/JB.187.11.3607-3619.2005 PMC1112053

[12] Kalscheuer R, Steinbuchel A (2003) A novel bifunctional wax ester synthase/acyl-CoA:diacylglycerol acyltransferase mediates wax ester and triacylglycerol biosynthesis in Acinetobacter calcoaceticus ADP1. J Biol Chem 278: 8075–8082. 12502715 10.1074/jbc.M210533200

[13] Steen EJ, Kang Y, Bokinsky G, Hu Z, Schirmer A, et al (2010) Microbial production of fatty-acid-derived fuels and chemicals from plant biomass. Nature 463: 559–562. 20111002 10.1038/nature08721

[14] Cheng JB, Russell DW (2004) Mammalian wax biosynthesis. I. Identification of two fatty acyl-Coenzyme A reductases with different substrate specificities and tissue distributions. J Biol Chem 279: 37789–37797. 15220348 10.1074/jbc.M406225200 PMC2757098

[15] Metz JG, Pollard MR, Anderson L, Hayes TR, Lassner MW (2000) Purification of a jojoba embryo fatty acyl-coenzyme A reductase and expression of its cDNA in high erucic acid rapeseed. Plant Physiol 122: 635–644. 10712526 10.1104/pp.122.3.635 PMC58898

[16] Reiser S, Somerville C (1997) Isolation of mutants of Acinetobacter calcoaceticus deficient in wax ester synthesis and complementation of one mutation with a gene encoding a fatty acyl coenzyme A reductase. J Bacteriol 179: 2969–2975. 9139916 10.1128/jb.179.9.2969-2975.1997 PMC179062

[17] Kaczmarzyk D, Fulda M (2010) Fatty acid activation in cyanobacteria mediated by acyl-acyl carrier protein synthetase enables fatty acid recycling. Plant Physiol 152: 1598–1610. 20061450 10.1104/pp.109.148007 PMC2832271

[18] Schirmer A, Rude MA, Li X, Popova E, del Cardayre SB (2010) Microbial biosynthesis of alkanes. Science 329: 559–562. 20671186 10.1126/science.1187936

[19] Winters K, Parker PL, Van Baalen C (1969) Hydrocarbons of blue-green algae: geochemical signfficance. Science 163: 467–468. 17731762 10.1126/science.163.3866.467

[20] Eser BE, Das D, Han J, Jones PR, Marsh EN (2011) Oxygen-independent alkane formation by non-heme iron-dependent cyanobacterial aldehyde decarbonylase: investigation of kinetics and requirement for an external electron donor. Biochemistry 50: 10743–10750. 22074177 10.1021/bi2012417 PMC3235001

[21] Li N, Norgaard H, Warui DM, Booker SJ, Krebs C, et al (2011) Conversion of fatty aldehydes to alka(e)nes and formate by a cyanobacterial aldehyde decarbonylase: cryptic redox by an unusual dimetal oxygenase. J Am Chem Soc 133: 6158–6161. 21462983 10.1021/ja2013517 PMC3113487

[22] Warui DM, Li N, Norgaard H, Krebs C, Bollinger JM Jr, et al (2011) Detection of formate, rather than carbon monoxide, as the stoichiometric coproduct in conversion of fatty aldehydes to alkanes by a cyanobacterial aldehyde decarbonylase. J Am Chem Soc 133: 3316–3319. 21341652 10.1021/ja111607x PMC3069495

[23] Li N, Chang WC, Warui DM, Booker SJ, Krebs C, et al (2012) Evidence for Only Oxygenative Cleavage of Aldehydes to Alk(a/e)nes and Formate by Cyanobacterial Aldehyde Decarbonylases. Biochemistry 51: 7908–7916. 22947199 10.1021/bi300912n

[24] Kelson TL, Secor McVoyJR, Rizzo WB (1997) Human liver fatty aldehyde dehydrogenase: microsomal localization, purification, and biochemical characterization. Biochim Biophys Acta 1335: 99–110. 9133646 10.1016/s0304-4165(96)00126-2

[25] Kok M, Oldenhuis R, van der Linden MP, Meulenberg CH, Kingma J, et al (1989) The Pseudomonas oleovorans alkBAC operon encodes two structurally related rubredoxins and an aldehyde dehydrogenase. J Biol Chem 264: 5442–5451. 2647719

[26] Lindahl R, Petersen DR (1991) Lipid aldehyde oxidation as a physiological role for class 3 aldehyde dehydrogenases. Biochem Pharmacol 41: 1583–1587. 2043148 10.1016/0006-2952(91)90157-z

[27] Liu ZJ, Sun YJ, Rose J, Chung YJ, Hsiao CD, et al (1997) The first structure of an aldehyde dehydrogenase reveals novel interactions between NAD and the Rossmann fold. Nat Struct Biol 4: 317–326. 9095201 10.1038/nsb0497-317

[28] Achterholt S, Priefert H, Steinbuchel A (1998) Purification and characterization of the coniferyl aldehyde dehydrogenase from Pseudomonas sp. Strain HR199 and molecular characterization of the gene. J Bacteriol 180: 4387–4391. 9721273 10.1128/jb.180.17.4387-4391.1998 PMC107445

[29] Vidal R, Lopez-Maury L, Guerrero MG, Florencio FJ (2009) Characterization of an alcohol dehydrogenase from the Cyanobacterium Synechocystis sp. strain PCC 6803 that responds to environmental stress conditions via the Hik34-Rre1 two-component system. J Bacteriol 191: 4383–4391. 19411329 10.1128/JB.00183-09 PMC2698509

[30] Perozich J, Nicholas H, Wang BC, Lindahl R, Hempel J (1999) Relationships within the aldehyde dehydrogenase extended family. Protein Sci 8: 137–146. 10210192 10.1110/ps.8.1.137 PMC2144113

[31] Mitchell DY, Petersen DR (1987) The oxidation of alpha-beta unsaturated aldehydic products of lipid peroxidation by rat liver aldehyde dehydrogenases. Toxicol Appl Pharmacol 87: 403–410. 3564015 10.1016/0041-008x(87)90245-6

[32] Nakayasu H, Mihara K, Sato R (1978) Purification and properties of a membrane-bound aldehyde dehydrogenase from rat liver microsomes. Biochem Biophys Res Commun 83: 697–703. 697851 10.1016/0006-291x(78)91045-8

[33] Kotchoni SO, Kuhns C, Ditzer A, Kirch HH, Bartels D (2006) Over-expression of different aldehyde dehydrogenase genes in Arabidopsis thaliana confers tolerance to abiotic stress and protects plants against lipid peroxidation and oxidative stress. Plant Cell Environ 29: 1033–1048. 17080931 10.1111/j.1365-3040.2005.01458.x

[34] De Laurenzi V, Rogers GR, Hamrock DJ, Marekov LN, Steinert PM, et al (1996) Sjogren-Larsson syndrome is caused by mutations in the fatty aldehyde dehydrogenase gene. Nat Genet 12: 52–57. 8528251 10.1038/ng0196-52

[35] Kirch HH, Nair A, Bartels D (2001) Novel ABA- and dehydration-inducible aldehyde dehydrogenase genes isolated from the resurrection plant Craterostigma plantagineum and Arabidopsis thaliana. Plant J 28: 555–567. 11849595 10.1046/j.1365-313x.2001.01176.x

[36] Sheehan J, Dunahay T, Benemann J, Roessler P (1998) A look back at the US Department of energy’s aquatic species program–:biodiesel from algae. National Renewable Energy Laboratory.

[37] Robertson DE, Jacobson SA, Morgan F, Berry D, Church GM, et al (2011) A new dawn for industrial photosynthesis. Photosynth Res 107: 269–277. 21318462 10.1007/s11120-011-9631-7 PMC3059824

[38] Chisti Y (2007) Biodiesel from microalgae. Biotechnol Adv 25: 294–306. 17350212 10.1016/j.biotechadv.2007.02.001

[39] Kaiser BK, Stoddard BL (2012) DNA recognition and transcriptional regulation by the WhiA sporulation factor. Sci Rep 1: 156. 10.1038/srep00156 PMC3240954 22355671

[40] Bustos SA, Golden SS (1991) Expression of the psbDII gene in Synechococcus sp. strain PCC 7942 requires sequences downstream of the transcription start site. J Bacteriol 173: 7525–7533. 1938947 10.1128/jb.173.23.7525-7533.1991 PMC212519

[41] Golden SS, Brusslan J, Haselkorn R (1987) Genetic engineering of the cyanobacterial chromosome. Methods Enzymol 153: 215–231. 3123881 10.1016/0076-6879(87)53055-5

[42] Andersson CR, Tsinoremas NF, Shelton J, Lebedeva NV, Yarrow J, et al (2000) Application of bioluminescence to the study of circadian rhythms in cyanobacteria. Methods Enzymol 305: 527–542. 10812624 10.1016/s0076-6879(00)05511-7

[43] Holtman CK, Chen Y, Sandoval P, Gonzales A, Nalty MS, et al (2005) High-throughput functional analysis of the Synechococcus elongatus PCC 7942 genome. DNA Res 12: 103–115. 16303742 10.1093/dnares/12.2.103

[44] Bligh EG, Dyer WJ (1959) A rapid method of total lipid extraction and purification. Can J Biochem Physiol 37: 911–917. 13671378 10.1139/o59-099

[45] Folch J, Lees M, Sloane Stanley GH (1957) A simple method for the isolation and purification of total lipides from animal tissues. J Biol Chem 226: 497–509. 13428781

[46] Bozzola JJ, Russell LD (1992) Electron Microscopy: Principles and Techniques for Biologists: Jones and Bartlett Publishers.

