# Supplementary material for: Fatty Aldehydes in Cyanobacteria Are a Metabolically Flexible Precursor for a Diversity of Biofuel Products
Source: PLoS One. 2013 Mar 11;8(3):e58307. doi: 10.1371/journal.pone.0058307 (PMC3594298; doi:10.1371/journal.pone.0058307)
Supplement: File S1 — Figure S1: Overexpression of Aar leads to a preferential accumulation of free fatty acids over alkanes. Samples were collected 24, 48 and 72 hours post-induction and analyzed by GC for total FAMES (a), pentadecane (b) and heptadecane (c). Figure S2: Purification of h6-AldE (orf0489). AldE containing an N-terminal 6XHis tag was expressed as a soluble protein in E. coli and purified by metal affinity and size exclusion chromatrography. The UV trace of the size exclusion chromatography step shows that AldE elutes at a size consistent with a homodimer (AldE is blue trace; green trace is of MW standards), as has been seen for other class 3 aldehyde dehydrogenases [27]. The inset is an SDS-PAGE gel, with the arrow indicating h6-AldE. Table S1: Plasmids used in these studies. (PDF) [file pone.0058307.s001.pdf]

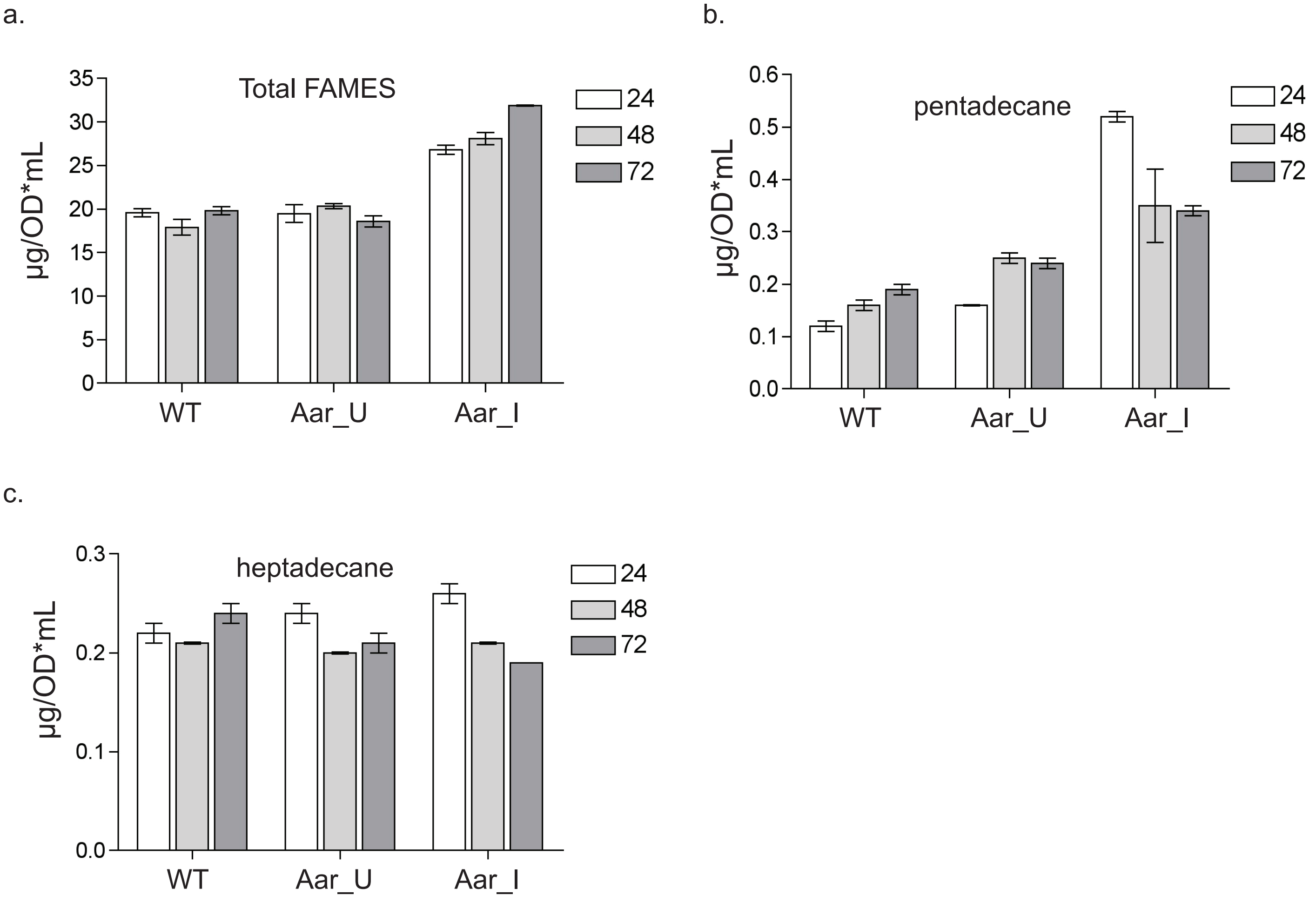

Supplementary Figure 1: Overexpression of Aar leads to a preferential accumulation of free fatty acids over alkanes. Samples were collected 24, 48 and 72 hours post-induction and analyzed by GC for total FAMES (a), pentadecane (b) and heptadecane (c).

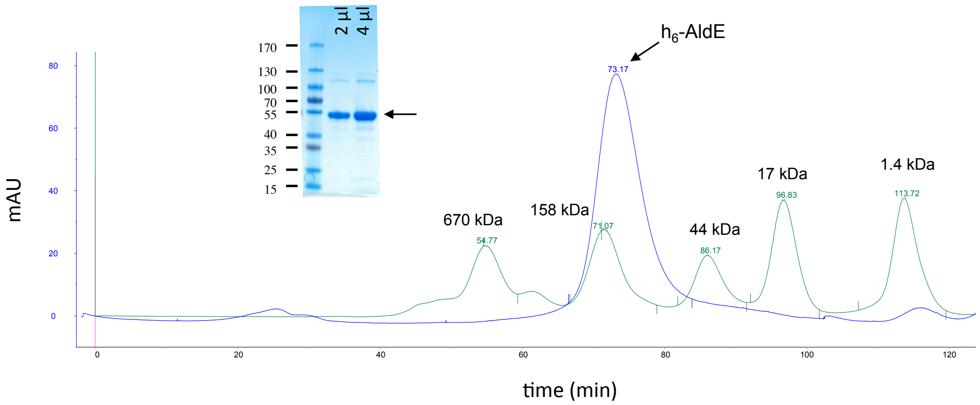

Figure S2: **Purification of h$_6$-AldE (orf0489)**

AldE containing an N-terminal 6XHis tag was expressed as a soluble protein in *E. coli* and purified by metal affinity and size exclusion chromatrography. The UV trace of the size exclusion chromatography step shows that AldE elutes at a size consistent with a homodimer (AldE is blue trace; green trace is of MW standards), as has been seen for other class 3 aldehyde dehydrogenases [27]. The inset is an SDS-PAGE gel, with the arrow indicating h$_6$-AldE.

Supplemental Table 1

Strains[1]	Description	Source or reference
<u>*S. elongatus* PCC 7942</u>	Wild-type strain	S.S. Golden
Aar (NS1)	An extra copy of Aar was inserted into NS1.	This study
Aar (NS2)	An extra copy of Aar was inserted into NS2.	This study
Aar (2X)	Aar was inserted into both NS1 and NS2	This study
ΔAldE	A chloramphenicol[r] cassette was inserted into bp 106-1259 of the AldE (orf0489) reading frame	This study
Aar(NS2)/ΔAldE	Aar was expressed from NS2 in a strain deleted for AldE.	This study
Aar (2X)/ AldE (NS4)	Aar was expressed from NS1 and NS2; and AldE was expressed from NS4	This study
ADGAT (NS4)	ADGAT from *A. baylyii* was expressed from NS4	This study
slr1192 (NS1)/Aar (NS2)/ADGAT[2]*(NS4)	slr1192 (alcohol dehydrogenase from Synechocystis PCC6803) was expressed from NS1; Aar from NS2; and ADGAT (*A. baylyii)* from NS4	This study
ACIAD3612 (NS1)/Aar (NS2)/ADGAT[2]*(NS4)	ACIAD3612 (alcohol dehydrogenase from *A. baylyii*) was expressed from NS1; Aar from NS2; and ADGAT (*A. baylyii)* from NS4.	This study
Plasmids		
pAM1579	Km[r], Ap[r]; NS2 recombination vector	S.S. Golden
pAM2314	Spec[r], NS1 recombination vector	S.S. Golden
pNS4	Gent[r]; NS4 recombination vector. Recombination arms are in the intergenic region between orfs 0893 and 0894.	This study

[1]All transgenes were expressed from the pTrc promoter
